# Incidence of AIDS-Defining Opportunistic Infections and Mortality during Antiretroviral Therapy in a Cohort of Adult HIV-Infected Individuals in Hanoi, 2007-2014

**DOI:** 10.1371/journal.pone.0150781

**Published:** 2016-03-03

**Authors:** Junko Tanuma, Kyu Ha Lee, Sebastien Haneuse, Shoko Matsumoto, Dung Thi Nguyen, Dung Thi Hoai Nguyen, Cuong Duy Do, Thuy Thanh Pham, Kinh Van Nguyen, Shinichi Oka

**Affiliations:** 1 AIDS Clinical Center, National Center for Global Health and Medicine, Tokyo, Japan; 2 Takemi Program in International Health, Harvard T.H. Chan School of Public Health, Boston, Massachusetts, United States of America; 3 Epidemiology and Biostatistics Core, The Forsyth Institute, Cambridge, Massachusetts, United States of America; 4 Biostatistics, Harvard T.H. Chan School of Public Health, Boston, Massachusetts, United States of America; 5 HIV Department, National Hospital of Tropical Disease, Hanoi, Vietnam; 6 Infectious Disease Department, Bach Mai Hospital, Hanoi, Vietnam; Infectious Disease Service, UNITED STATES

## Abstract

**Background:**

Although the prognosis for HIV-infected individuals has improved after antiretroviral therapy (ART) scale-up, limited data exist on the incidence of AIDS-defining opportunistic infections (ADIs) and mortality during ART in resource-limited settings.

**Methods:**

HIV-infected adults in two large hospitals in urban Hanoi were enrolled to the prospective cohort, from October 2007 through December 2013. Those who started ART less than one year before enrollment were assigned to the survival analysis. Data on ART history and ADIs were collected retrospectively at enrollment and followed-up prospectively until April 2014.

**Results:**

Of 2,070 cohort participants, 1,197 were eligible for analysis and provided 3,446 person-years (PYs) of being on ART. Overall, 161 ADIs episodes were noted at a median of 3.20 months after ART initiation (range 0.03–75.8) with an incidence 46.7/1,000 PYs (95% confidence interval [CI] 39.8–54.5). The most common ADI was tuberculosis with an incidence of 29.9/1,000 PYs. Mortality after ART initiation was 8.68/1,000 PYs and 45% (19/45) died of AIDS-related illnesses. Age over 50 years at ART initiation was significantly associated with shorter survival after controlling for baseline CD4 count, but neither having injection drug use (IDU) history nor previous ADIs were associated with poor survival. Semi-competing risks analysis in 951 patients without ADIs history prior to ART showed those who developed ADIs after starting ART were at higher risk of death in the first six months than after six months.

**Conclusion:**

ADIs were not rare in spite of being on effective ART. Age over 50 years, but not IDU history, was associated with shorter survival in the cohort. This study provides in-depth data on the prognosis of patients on ART in Vietnam during the first decade of ART scale-up.

## Introduction

Antiretroviral therapy (ART) has resulted in a remarkable decline in acquired immunodeficiency syndrome (AIDS)-related death among HIV-infected individuals worldwide [1–6]. As prognosis has improved, reports from resource-rich countries have shown that the causes of death in HIV-infected individuals have changed, with cancers or cardiovascular diseases or liver-related diseases becoming the leading causes of mortality. [7–10].

Although a detailed understanding of causes of death and associated risk factors is crucial to the appropriate management of HIV-related diseases and co-morbidities, the specific causes of death have not been well described in resource-limited settings. Additionally, all-cause mortality of HIV-infected individuals is still higher in resource-limited than resource-rich countries [2]. Despite the high efficacy of ART, opportunistic infections (OIs) can develop while the patient is on ART, either due to the unmasking of subclinical infection that occurs with immune recovery, or due to prolonged immunosuppression. Treatment failure also facilitates the development of OIs at any time during ART. As a result, AIDS-defining illnesses (ADIs) have remained major morbidities in HIV-infected individuals in resource-limited settings, even in the era of ART [11–13]. Furthermore, previous reports have shown high mortality rates among injection drug users (IDUs) from drug overdose, suicide, accidents, violence, or liver-related diseases [14, 15]. In Vietnam, where a large part of the HIV epidemic has been driven by IDUs, the mortality rate among IDUs with or without HIV infection was reported to be as much as 13-fold higher than that in the general population [16]. Thus, the overall prognosis of HIV-infected individuals in Vietnam may partly reflect the social and epidemiological characteristics of IDUs. However, few studies have addressed the incidence of AIDS, mortality, or specific causes of death in HIV-infected individuals receiving ART in Vietnam [17].

In this prospective cohort study of HIV-infected adults on ART in two large hospitals in urban Hanoi, Vietnam, we aimed to describe the incidence of ADIs, specific causes of death, mortality rates, and risk factors associated with the development of ADIs and shorter survival time, from 2007 through 2014.

## Methods

### Study Population and Data Collection

A prospective cohort study of HIV-infected adults was conducted in two large hospitals in urban Hanoi, Vietnam: Bach Mai Hospital (BMH) and the National Hospital of Tropical Diseases (NHTD). Patients attending the two HIV clinics were recruited from April 2011 through October 2012 in BMH and from 2007 to 2013 in NHTD by contacting all who were on ART. Participants were enrolled after providing written informed consent as set out in the study protocol approved by the ethics committee and the institutional ethical review boards. Participants in the cohort had different histories with respect to ART prior to enrollment. We excluded from the present analysis those who had received ART for more than one year prior to enrollment. Information was obtained on ADIs that occurred before and after ART, non-ADI clinical events, medication and laboratory data using standardized forms at enrollment and at each follow-up visit scheduled six-monthly until the end of April 2014. The causes of death were classified according to the Coding of Causes of Death in HIV (CoDe) [18]. The Center for Disease Control’s (CDC) list for AIDS-defining illnesses [19] was used for coding all ADIs except wasting syndrome, for which it was difficult to determine the date of onset. However, wasting syndrome was used in the classification of causes of death as an AIDS-related death [18]. A second or third episode of tuberculosis (TB) in a same person was counted as a new event if it occurred after completing treatment and if more than a year had passed since the diagnosis of a previous TB episode. Diagnosis, prophylaxis, treatment of OIs, and ART were based on the Vietnamese national guidelines [20–22], which were updated twice during the study period according to updates in the World Health Organization (WHO) ART guidelines [23, 24]. The CD4 count for ART indication changed from 200/mm^3^ to 250/mm^3^ in 2009 [21] and to 350/mm^3^ in 2011. Zidovudine (AZT) or stavudine (d4T) was replaced with tenofovir (TDF) in the preferred first-line regimen in 2011 [22]. Follow-up was censored when patients were transferred to other hospitals or lost from clinical care in the study sites for more than 12 months.

The study protocol was approved by the ethics committee in the Vietnamese Ministry of Health (No:1666/QD-BYT) and the institutional ethical review boards in BMH, NHTD and the National Center for Global Health and Medicine (NCGM) in Tokyo, Japan (NCGM-G-001074-01).

### Statistical Analysis

Incidence rate of ADIs were calculated by dividing the number of patients who developed an event by the number of person-years (PYs) on ART. In order to identify the factors for incidence of ADIs, we fitted a Poisson regression model. We estimated the effects of potential risk factors on all-cause mortality by fitting a Cox proportional hazards model. These analyses were conducted by including one covariate at a time (univariate analysis) or all covariates at the same time (multivariable analysis) into the regression models. In addition, we estimate survival functions for ADIs and death using joint semi-competing risks analysis [25, 26] to investigate the impact of developing ADIs on occurrence of subsequent deaths among those without a history of ADI before ART. Such analyses make explicit use of information on the timing of death following ADI, which would be ignored in a traditional competing risks analysis [27]. This, in-turn, permitted the investigation of how the risk of death changed over time, depending on whether a new ADI event occurred. We computed the explanatory hazard ratio, defined by the ratio of the risk of death with and without a new ADI at any given point in time [26]. For comparison, we also presented results from univariate Weibull regression analyses of ADIs and death. For all statistical analyses, differences were considered significant if the p value was less than 0.05. Analyses were performed using STATA version 12 (StataCorp LP, TX, U.S.A.) and R Statistical Software version 3.2.0 (Foundation for Statistical Computing, Vienna, Austria) [28].

## Results

### Characteristics of the Study Population

In total, 2,070 individuals were enrolled to the cohort from October 2007 until the end of 2013. Of those, 190 who had never received ART and 683 who had been on ART for more than a year at enrollment were excluded from the analysis. The remaining 1,197 were assigned to the present analysis, and contributed to 3,446 PYs. Of these, 951 had not developed ADIs at the time of starting ART; they contributed 2,763 PYs. The characteristics of the participants are shown in Table 1. Overall, 63% of the study participants were men and the median age was 32 years. Sexual contact was reported as a possible route of infection in 74% participants; 28% declared previous injection drug use; while the proportion of hepatitis C (HCV) coinfection, which strongly indicates possible multiple-needle-sharing exposure, was 42%. Twenty participants had other possible risk factors, including tattooing, accidents that happened while medical care was being administered, or receiving blood products. Of these participants, three also reported sexual contact as a possible route of infection. Sixty-six (5.5%) refused to answer or did not answer questions on HIV risk factors. The majority of patients with injection drug use experience were men and the percentage of IDUs was considerably greater in men than in women (44.5% in men vs. 1.3% in women; p<0.001). ART was started a median of 2 months after HIV diagnosis at a median CD4 count of 110/mm3. The median CD4 count at baseline increased significantly after the Vietnamese guideline on ART indication were changed in 2011 (median 75/mm^3^ before 2011 vs. 269/mm^3^ after 2011; p<0.001). According to these guidelines, the majority of the participants started ART regimens that included a nucleos(t)ide reverse transcriptase inhibitors (NRTI) backbones of either AZT plus lamivudine (3TC), d4T plus 3TC or TDF plus 3TC (48.1%, 26.6%, and 24.6%, respectively) plus the non-nucleoside reverse-transcriptase inhibitor (NNRTI) nevirapine (NVP) or efavirenz (EFV)– 45.8% and 53%, respectively. Those who experienced ADIs before ART were less likely to have AZT plus 3TC and more likely to have TDF plus 3TC as an NRTI backbone and EFV as the third drug in their initial ART regimens. Those eligible for analysis were enrolled to the cohort at a median of 5.2 months after ART initiation: 678 (56.6%) joined the cohort within the first six months of ART. Of 1,195 participants who had HIV viral load results available for at least two time points, 1,141 (95.5%) had achieved viral suppression, at less than 200 copies/ml, prior to censoring. Compared to those without any ADI episodes before ART, participants who had experienced ADIs before starting ART were older, had lower CD4 counts at baseline, and had started ART in an earlier calendar year. In addition, a significantly greater proportions were men, IDUs or had tested positive for anti-HCV antibodies (Table 1).

**Table 1:** Characteristics of Study Participants.

			ADIs at baseline				
	Total (n = 1,197)		Yes (n = 246)		No (n = 951)		*p*
Age at starting ART, median (range)	32	(18–73)	33	(19–73)	32	(18–70)	<0.001
Gender male, n (%)	750	(62.7)	195	(79.3)	555	(58.4)	<0.001
HIV risk factor							
Sexual contact, n (%)	890	(74.4)	174	(70.7)	716	(75.3)	0.20
Injection drug use, n (%)	340	(28.4)	101	(41)	239	(25.1)	<0.001
Other/unknown, n (%)	91	(7.6)	14	(5.7)	77	(8.1)	0.36
HBs antigen positive, n (%)^a^	161	(13.5)	31	(12.6)	132	(14.3)	0.40
Anti-HCV antibody positive, n (%)^a^	441	(42.0)	124	(50.4)	317	(39.5)	<0.001
CD4 count at baseline, median (range)^a^	110	(1–693)	42	(1–550)	144	(1–693)	<0.001
Initial ART regimen, NRTI							
AZT+3TC/FTC	576	(48.1)	93	(37.8)	483	(50.8)	<0.001
d4T+3TC/FTC	318	(26.6)	72	(29.3)	246	(25.9)	0.32
TDF+3TC/FTC	294	(24.6)	81	(32.9)	213	(22.4)	0.001
Others	9	(0.8)	0		9	(0.9)	
Initial ART regimen, third drug							
NVP	548	(45.8)	62	(25.2)	486	(51.1)	<0.001
EFV	635	(53.0)	183	(74.4)	452	(47.5)	<0.001
Others	14	(1.2)	1	(0.4)	13	(1.4)	
Time from ART start to enrollment, median months (range)	5.2	(0–12)	4.6	(0–12)	5.4	(0–7.9)	0.20
Time from HIV diagnosis to ART, median months (range)	2.0	(0–211)	1.2	(0–135)	2.3	(0–211)	<0.001
Time on ART, median months (range)	32	(0.6–91)	30	(0.6–91)	33	(1–91)	0.10

Note:

^a^–Data were unavailable in 20 subjects for HBs antigen, in 149 for Anti-HCV antibody and in 167 for CD4 count at baseline.

ADI: AIDS-defining illness; NRTI: nucleoside reverse transcriptase inhibitor; AZT zidovufine; 3TC: lamivudine; FTC: emtricitabine; d4T: stavudine; TDF: tenofovir; NVP: nevirapine; EFV: efavirenz

### Incidence of AIDS-Defining Opportunistic Infections and Risk Factors

**Fig 1:**
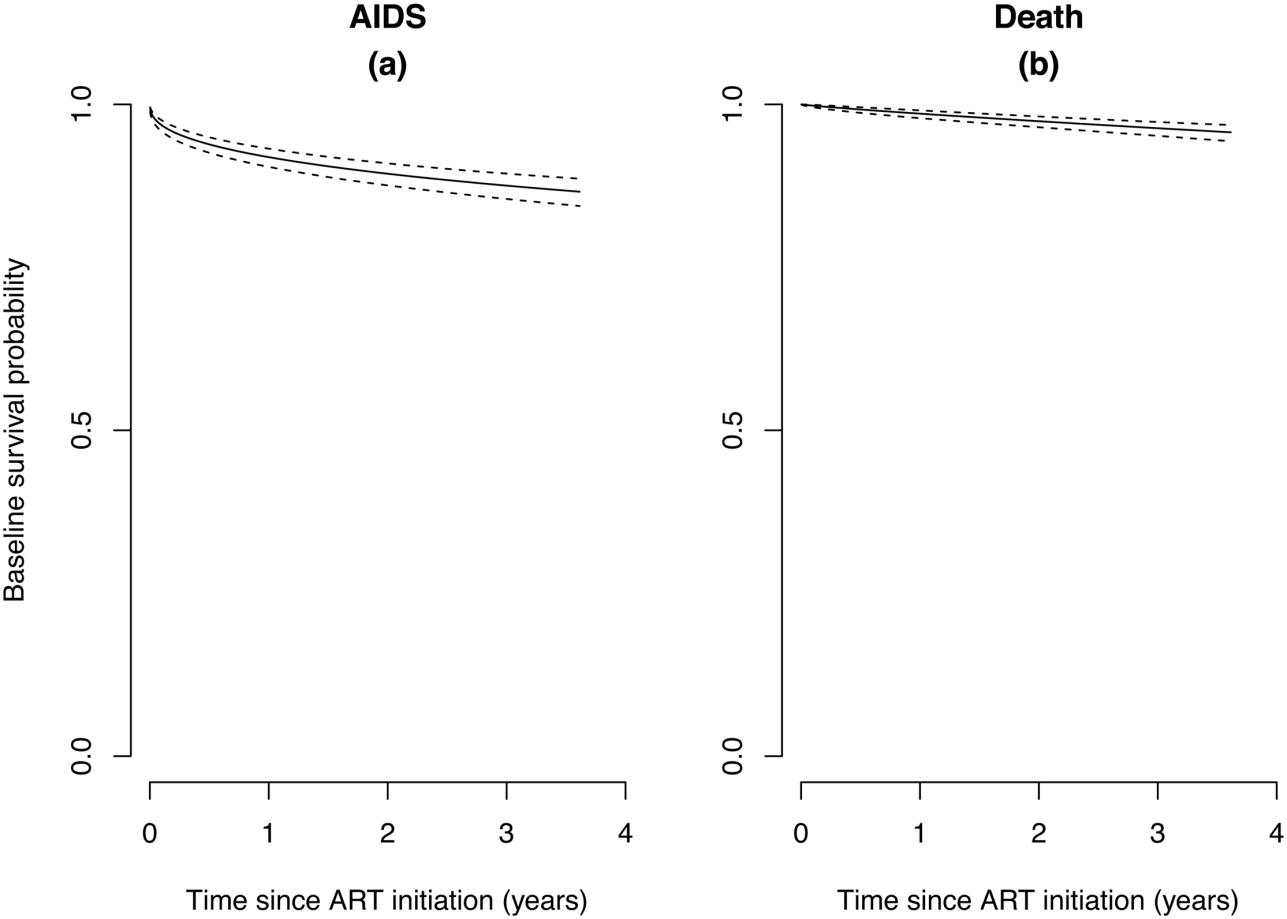
Survival Probabilities on ART in All Study Participants. (a) Survival without new AIDS events on ART. (b) Overall survival on ART. Dotted lines indicate ranges of 95% CIs.

**Fig 2:**
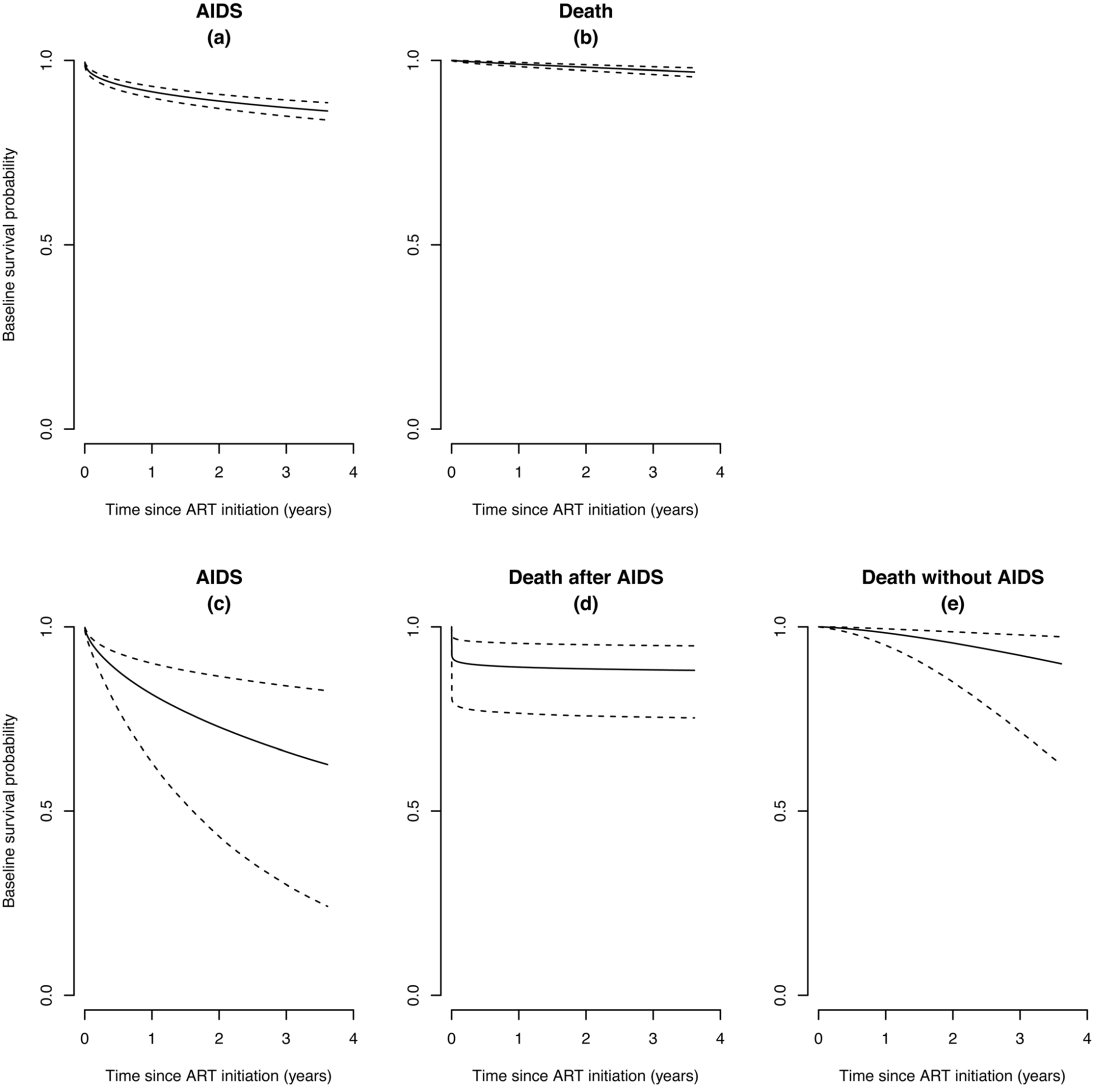
Survival Probabilities on ART in Subjects without AIDS History before ART. (a) Survival without AIDS on ART in Weibull regression analysis. (b) Overall survival on ART in Weibull regression analysis. (c) Survival without AIDS on ART in semi-competing risks analysis. (d) Time to death without acquiring AIDS during ART in semi-competing risks analysis. (e) Time to death following new AIDS events during ART in semi-competing risks analysis. Dotted lines indicate ranges of 95% CIs.

Estimated probabilities of participants not developing new ADIs, and estimated overall survival probabilities after starting ART are illustrated in Fig 1a and 1b for all 1,197 participants and in Fig 2a and 2b for the 951 without previous ADIs before ART, respectively. In total, 161 episodes of ADIs were observed after starting ART in 137 patients (46.7/1000PYs, 95% confidence interval [CI] 39.8–54.5) at a median of 3.2 months on ART (range 0.03–75.8). The number of episodes and the incidence rate of each ADI are shown in Table 2. The most common ADI was TB (28.4/1,000 PYs, 95% CI 23.1–34.7), followed by toxoplasmosis and pneumocystis pneumonia.

**Table 2:** Incidence Rates of AIDS-Defining Opportunistic Infections during ART.

AIDS-defining illnesses (ADIs)	Number of episodes, Incidence Rate (/1000 PYS), (95%CI)					
	All (n = 1,197; 3,446 person-years)			No prior ADIs at baseline (n = 951; 2,763 person-years)		
Any ADIs	161	46.7	(39.8–54.5)	130	47.1	(39.3–55.9)
Tuberculosis	98	28.4	(23.1–34.7)	79	28.6	(22.6–35.6)
Toxoplasmosis of brain	16	4.64	(2.65–7.54)	14	5.01	(2.77–8.50)
Pneumocystis pneumonia	10	2.90	(1.39–5.34)	8	2.89	(1.25–5.70)
Esophageal candidiasis	7	2.03	(0.82–4.19)	7	2.53	(1.02–5.22)
Cytomegalovirus retinitis	6	1.74	(0.64–3.79)	6	2.17	(0.80–4.72)
*Mycobacterium avium* complex	6	1.74	(0.64–3.79)	4	1.45	(0.39–3.71)
Recurrent bacterial pneumonia	5	1.45	(0.47–3.39)	4	1.45	(0.39–3.71)
Cryptococcus	4	1.16	(0.32–2.97)	4	1.45	(0.39–3.71)
Cervical cancer	2	0.58	(0.070–2.10)	2	0.72	(0.088–2.61)
Progressive multifocal leukoencephalopathy	2	0.58	(0.070–2.10)	0	-	-
Disseminated HSV infection	2	0.58	(0.070–2.10)	0	-	-
Primary CNS lymphoma	1	0.29	(0.007–1.62)	0	-	-
Non-Hodgkin’s lymphoma	1	0.29	(0.007–1.62)	1	0.36	(0.009–2.01)
Salmonella septicemia	1	0.29	(0.007–1.62)	1	0.36	(0.009–2.01)

CNS: central nervous system

From the univariate analyses, male gender, injection drug use and anti-HCV-antibody positivity were significantly associated with higher risk of developing new ADIs, while the multivariable analyses showed only age older than 50 years old and CD4 counts less than 200 /mm^3^ at baseline to be significant among the 951 patients without ADI history at baseline (Table 3). ADIs prior to ART was not statistically associated with the risk of acquiring new ADIs.

**Table 3:** Factors Associated with Acquiring AIDS-Defining Opportunistic Infections during ART.

Variables	Univariate models, IRR (95%CI), *p*						Multivariate models, IRR (95%CI), *p*					
	All (n = 1,197)			No prior ADIs at baseline (n = 951)			All (n = 1,197)			No prior ADIs at baseline (n = 951)		
Gender male	2.23	(1.52–3.25)	<0.001*	2.80	(1.83–4.28)	<0.001*	1.47	(0.85–2.56)	0.19	1.86	(0.97–3.57)	0.06
Age 20–29	1						1			1		
Age 30–39	1.32	(0.91–1.90)	0.14	0.89	(0.37–2.17)	0.804	1.34	(0.82–2.20)	0.31	1.40	(0.80–2.44)	0.24
Age 40–49	1.29	(0.75–2.20)	0.36	1.32	(0.42–4.16)	0.636	1.60	(0.84–3.04)	0.15	1.59	(0.75–3.37)	0.23
Age over 50	1.46	(0.80–2.67)	0.22	0.91	(0.24–3.52)	0.892	1.83	(0.81–4.14)	0.15	2.46	(1.01–5.95)	0.046*
Injection drug use	1.71	(1.25–2.34)	0.001*	1.93	(1.36–2.74)	<0.001*	1.31	(0.75–2.30)	0.34	1.15	(0.61–2.16)	0.68
HBs antigen positive	1.31	(0.87–1.98)	0.19	1.43	(0.92–2.23)	0.114	1.28	(0.75–2.16)	0.36	1.28	(0.70–2.35)	0.43
Anti-HCV antibody positive	1.83	(1.31–2.55)	<0.001*	2.25	(1.55–3.27)	<0.001*	1.19	(0.68–2.10)	0.55	1.54	(0.81–2.92)	0.19
CD4<200 at baseline	9.73	(3.98–23.9)	<0.001*	5.70	(2.32–14.0)	<0.001*	4.42	(3.10–19.1)	<0.001*	6.94	(2.74–17.0)	<0.001*
Prior ADIs at baseline	0.92	(0.62–1.36)	0.68	-			0.69	(0.43–1.10)	0.12	-		

IRR: incident rate ratio; CI: confidential interval; ADI: AIDS-defining illness; HBs antigen: hepatitis B surface antigen; HCV: hepatitis C virus

Note:

* Statistically significant (P<0.05)

### Mortality and Causes of Deaths

In total, 42 (3.5%) participants died after median 1.13 years on ART (range 0.05–7.27 years) with median CD4 count 126/mm^3^ (range 3–479/mm^3^) at death, giving a mortality rate of 11.9/1,000 PYs (95% CI 8.78–16.5). Nineteen participants who died (45.2%) had viral loads of less than 200 copies/ml. Of the 33 patients for whom the causes of death was successfully identified, 19 died due to ADIs and 14 died due to other causes (Table 4). There was no significant differences in survival time on ART between the 19 AIDS-related deaths and 14 non-AIDS deaths (median of 1.05 years in AIDS-related deaths vs. 1.61 years in non-AIDS deaths; p = 0.29).

**Table 4:** Causes of Deaths.

Causes of Deaths, n (%)	All		Prior ADIs at baseline			
			Yes		No	
Total	42		18		24	
AIDS-defining illnesses (ADIs)	19	(45.2)	9	(50)	10	(41.7)
TB	7		4		3	
Wasting Syndrome	6		2		4	
PML	2		2		0	
Pneumonia	2		1		1	
Cervical cancer	1		0		1	
Toxoplasma	1		0		1	
Others	14	(33.3)	4	(22.2)	10	(41.7)
Renal failure	4		1		3	
Cancer (not cervical cancer)	2		1		1	
Stroke	2		0		2	
Influenza H1N1	2		1		1	
*Penicillium marneffei* infection	1		0		1	
Liver failure	1		0		1	
Accident	1		0		1	
Drug overdose	1		1		0	
Not identified	9	(21.5)	5	(27.8)	4	(16.6)

ADI: AIDS-defining illness; TB: tuberculosis; PML: progressive multifocal leukoencephalopathy

**Fig 3:**
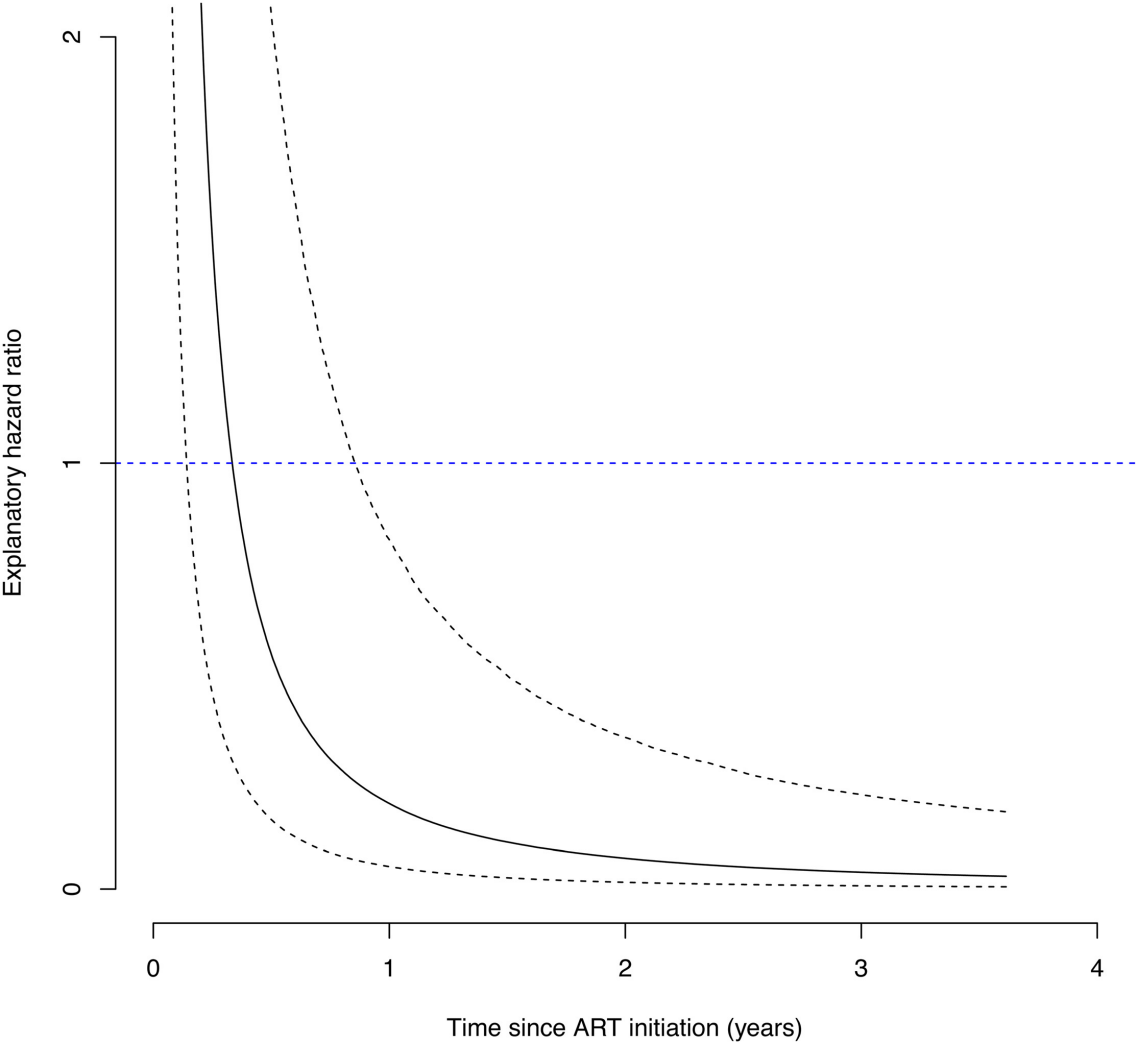
Explanatory Hazard Ratio in Semi-Competing Risks Analysis. The ratio of hazards for death after and before AIDS. If the risk of death is not influenced by the risk of AIDS (i.e., time to AIDS and death are independent) the ratio is equal to one (the blue horizontal line). Dotted lines indicate ranges of 95% CIs.

The Cox proportional hazards model showed that male gender, older age, injection drug use, baseline CD4 less than 200/mm^3^, and ADIs prior to ART were associated with shorter survival in univariate models, while only older age (over 50 years old in all 1,197 patients and over 40 years old in 951 patients without ADIs prior to ART) was significant in multivariate models (Table 5). In the semi-competing risks analysis of the 951 patients without ADIs prior to ART, deaths following new ADI events appeared to be more likely to occur within a short period after starting ART (Fig 2e), while the risk of deaths without acquiring ADIs showed a gradual increase overtime (Fig 2d). In Fig 3, we see that the explanatory hazard ratio is substantially larger than 1 immediately after ART initiation, implying that the development of the ADIs considerably increases the risk of death at the beginning of ART. Subsequently, however, the explanatory hazard ratio rapidly declined and the risk of death without acquiring ADIs outweighed that following ADIs after 4.1 months of ART.

**Table 5:** Factors Associated with All-Cause Mortality.

Variables	Univariate models, HR (95%CI), *p*						Multivariate models, HR (95%CI), *p*					
	All (n = 1197)			No prior ADIs at baseline (n = 951)			All (n = 1197)			No prior ADIs at baseline (n = 951)		
Gender male	3.61	(1.52–8.57)	0.004*	8.02	(1.89–34.1)	0.005*	1.14	(0.37–3.51)	0.82	5.52	(0.64–47.4)	0.12
Age 20–29	1			1			1			1		
Age 30–39	1.69	(0.74–3.83)	0.21	2.76	(0.77–9.92)	0.12	1.16	(0.43–3.13)	0.78	1.85	(0.36–9.60)	0.47
Age 40–49	2.5	(0.87–7.22)	0.09	5.23	(1.17–23.4)	0.03*	2.69	(0.85–8.55)	0.09	5.91	(1.06–32.9)	0.043*
Age over 50	4.53	(1.69–12.1)	0.003*	11.0	(2.74–43.9)	0.01*	7.39	(1.90–28.8)	0.004*	11.7	(1.75–77.6)	0.011*
Injection drug use	2.00	(1.09–3.67)	0.026*	1.38	(0.59–3.23)	0.46	1.76	(0.61–5.09)	0.30	0.93	(0.19–4.55)	0.93
HBs antigen positive	1.48	(0.69–3.20)	0.32	2.54	(1.05–6.12)	0.38	1.72	(0.69–4.26)	0.24	2.48	(0.81–7.60)	0.11
Anti-HCV antibody positive	1.80	(0.96–3.39)	0.07	1.24	(0.54–2.83)	0.61	1.99	(0.60–6.54)	0.26	1.79	(0.39–8.21)	0.46
CD4<200 at baseline	9.64	(1.31–70.8)	0.026*	5.95	(0.78–45.3)	0.85	6.12	(0.81–46.3)	0.08	5.12	(0.66–39.9)	0.12
Prior ADI at baseline	2.96	(1.60–5.46)	0.001*	-			2.03	(0.96–4.27)	0.06	-		

HR: hazard ratio; CI: confidential interval; ADI: AIDS-defining illness; HBs antigen: hepatitis B surface antigen; HCV: hepatitis C virus

Note:

* Statistically significant (P<0.05)

## Discussion

In this study, we described the incidence of ADIs and mortality after ART initiation in a cohort of HIV-infected adults in two large hospitals in urban Hanoi, Vietnam. Although 11.4% of study participants developed ADIs after starting ART, the overall mortality rate was low, irrespective of ADI history prior to ART. Almost half the deaths were attributable to ADIs and older age at baseline was statistically associated with shorter survival. IDU history was not a significant factor for developing ADIs or for increased mortality. This study provides in-depth data on the prognosis of HIV-infected individuals who started ART in Vietnam, where there has been the extensive ART scale-up over the last decade.

The overall incidence of ADIs during ART was as high as 46.1/1,000PYs in this cohort. There have been a few reports on the incidence of OIs during ART in HIV-infected individuals thus far [11–13], one of which was from the Swiss HIV Cohort Study, reporting 36/1,000PYs [29]. The ADI incidence is largely influenced by regional factors, such as endemic levels of specific opportunistic infections, the ART indication in the regional guidelines, and accessibility of healthcare. A possible explanation for the higher ADI incidence in our cohort (vs. the Swiss cohort) is the more severe immunodeficiency at baseline and the different OI distribution in our cohort, particularly the higher incidences of TB, toxoplasmosis and cryptococcal meningitis, none of which are common in resource-rich countries. Conversely, a study from Thailand had reported a substantially higher incidence of ADIs on ART (82/1,000PYs) than our results [13]. Although the pattern of OIs was similar to the Thai study, our patients might have been recruited to the study at an earlier clinical stage. Furthermore, more patients were initiated on ART after the WHO guideline increased the recommended CD4 count for ART indication from 200 to 350/mm^2^ in 2010 [24] and thus had remarkably higher baseline CD4 counts than patients in the Thai study. Importantly, this change in CD4 counts for ART indication significantly increased the median baseline CD4 count, leading to a decline in ADI incidence in our cohort. Since immune status at ART initiation is one of the most crucial factors determining the incidence of ADIs during ART, earlier treatment should continue to be promoted to reduce morbidity of patients on ART.

Comparing mortality rates between different studies is challenging because of differences in selection criteria and socio-demographic backgrounds of participants. A cohort study of 894 IDUs in Thai Nguyen province in Northern Vietnam showed that the mortality of HIV-negative and HIV-positive persons were 41/1,000 PYs and 146/1,000 PYs respectively [16]. Based on this report, we assumed that the high proportion of IDUs—who are vulnerable for liver-related death, suicide, accidents or drug overdose [14, 15]—might lead to a high morality in our study population. Surprisingly, however, the overall mortality was 8.68/1,000 PYs and the mortality among IDUs was 18.9/1,000PYs, comparable to those in Western countries (range 10.4–20.1/1,000) [9]. A possible reason for the low mortality in our study may be the better immune status at baseline. Although acquiring ADIs was not rare despite being on ART, the fatality of acquired ADIs was low. In addition, plasma viral load monitoring was performed free of charge 6-monthly intervals and changes were made to ART regimens according to these results based on the national ART guideline. This may have contributed to the high proportion of virologic success of ART and may have led to a reduction in AIDS-related deaths. It may have also played some part inreduction in the frequency of non-AIDS deaths [30]. In addition, most of our patients had taken cotrimoxazole as prophylaxis as indicated by the national guideline. This is widely acknowledged to reduce the incidence and mortality of AIDS-related conditions [31]. Another possible factor for better prognosis may be the characteristics of the study sites; both study sites were referral hospitals in urban settings, with more highly qualified staff and more advanced facilities than other clinics in Vietnam. Furthermore, the study sites made extensive efforts to actively trace of patients who had missed their scheduled visits, either by healthcare professionals or by peer supporters. We believe this led to the high rate of retention in care, 95.3% at 12 months of ART [32] and, as a result, the high proportion of virologic suppression (95.5%). Notably, having an IDU history was not statistically associated with increased mortality. We speculate that the extensive effort to keep patients retained in care might have facilitated communication between patients and healthcare professionals leading to an effective reduction in non-AIDS-related deaths, such as suicide, involvement to violence and accidents or drug overdose, all of which are common in IDUs.

This is the first study to describe causes of death in patients receiving ART in Vietnam. Although the accurate diagnosis of cause of death is always challenging in resource-limited settings, we collected as much clinical information as possible and successfully identified the underlying causes of death in all hospitalized cases. We found that 45% of deaths were attributable to AIDS, while non-AIDS illnesses were responsible for 33% of deaths. Although the size of our cohort was small, the proportion of AIDS-related deaths among all deaths was comparable to those reported by previous large studies (29% to 50%) [4, 7, 9]. Like other low- and middle-income countries, TB was the most common ADI seen in 8.2% in this study; this incidence is similar to previous studies [13, 33, 34]. However, the case-fatality rate of TB in our cohort (7.1%) is low compared with other resource-limited settings (7.0 to 27.4%) [33, 34]. Since all our TB cases were in the ART program, they may have been diagnosed and treated in the early stages of TB, which might have led to low fatality. On the other hand, although several studies have highlighted the increasing importance of liver-related deaths [35], non-AIDS malignancies and cardiovascular diseases [9], the rarity of these events in this study made it difficult to evaluate whether these conditions would have increased in this setting. Notably, there was only one confirmed case of liver-related death, despite the high prevalence of hepatitis B or C co-infection. However, there were 9 cases (21.5%) whose causes of death were unidentifiable because they died at home. Although almost impossible to know if their deaths were related to injection drug use or other socially undesirable behaviors that could have prevented them from accessing care when dying, we need to view the distributions of causes of death in the context of such limitations of unidentifiable causes of deaths.

We found that age older than 50 years was associated with higher ADI incidence after controlling for CD4 count in 951 patients without ADI episodes prior to ART. Additionally, age older than 40 or 50 years was related to higher mortality during the observation period of up to 7.5 years. Estimated life expectancy in the Vietnamese general population is 73 years old [36], far beyond the maximum age of our patients, and half the deaths in our study were attributable to ADIs. Thus, the increased risk of shorter survival in older patients might not solely be explained by aging-associated conditions occurring independently of HIV infection. This finding is compatible with previous studies reporting that ART initiation at older ages is related to higher mortality, as a consequence of delayed HIV diagnosis and initiation of ART compared with younger patients [37]. Although not significant in multivariate models, the incidence of ADIs and mortality was higher in men than those in women in univariate models with high hazard ratios (2.23–8.02). Similarly, there have been a number of previous reports on gender and ART outcomes from resource-limited countries; most revealed higher mortality in men than women on ART [38–40]. It is usually speculated that such poorer survival in men is explained by late ART initiation due to their poorer health-seeking behavior and increased likelihood of their loss to follow up or treatment failure. Therefore, it is reasonable to view both older age and male gender as risk factors for shorter survival. Those having such factors should be carefully followed-up, as should IDUs.

To explore the impact of developing ADIs on survival, we evaluated whether experiencing an ADI prior to ART increased the risk of all-cause mortality after ART initiation. We found a significantly higher mortality after acquiring ADIs on ART in univariate—but not in multivariate—models. Patients without ADI events prior to ART were assigned to semi-competing risks analysis, where we found that death after acquiring ADIs was more likely to occur within six months of ART initiation, but rapidly decreased thereafter. Interestingly, however, survival time did not differ by cause of death (AIDS-deaths vs. non-AIDS deaths). Although this result was not conclusive and needs to be viewed within the limitations of statistical power due to the small sample size and the rarity of deaths, it implies that those who experienced ADIs might be more vulnerable to high mortality in the first six months of ART. Thus, early ART initiation, to prevent acquiring ADIs after starting ART, might be key to reducing the risk of shorter survival.

In addition to the small sample size and the rarity of fatal events, our study had several limitations. First, the duration of ART was diverse at study enrollment, particularly at the beginning of the study. Thus, we might have failed to include patients who died within the first 12 months of ART before 2007. This could make the survival time longer and the results of regression analysis could be biased toward null. Second, the accuracy of ADI diagnosis could have been affected by limitations of resource availability; ADI may have been underdiagnosed. In fact, examinations for opportunistic infections are not covered by ART programs, which may have discouraged patients to undergo investigation for financial reasons. Third, since our study clinics were both in large referential hospitals in urban Hanoi, it is unclear to what extent our results could be generalized. Strengths of our study include the prospective collection of events using structured reporting forms and the high retention rate in care by the active patient tracing system. Usually, ascertainment of deaths among people lost to follow-up is a critical problems in survival analysis in resource-limited settings. The active patient tracing system in our study sites enabled us to minimize censoring due to loss from the care.

In conclusion, the mortality after ART initiation in two large hospitals in urban Hanoi was comparable to that of Western countries, and AIDS-defining opportunistic infections were not rare while on ART. Almost half the deaths were attributable to AIDS, and age over 50 years at ART initiation was statistically associated with shorter survival, while IDU history was not associated with poor prognosis. This is the first in-depth data on survival of HIV-infected patients receiving ART, and provides supportive evidence for the success of ART programs in urban Hanoi in the decade of rapid ART scale-up.
